# Myosteatosis Predicts Poor Prognosis in Patients With Castration‐Resistant Prostate Cancer Treated With Enzalutamide

**DOI:** 10.1111/iju.70476

**Published:** 2026-04-29

**Authors:** Tomohiro Kanaki, Koji Hatano, Toshiki Oka, Masaru Tani, Masatoshi Konishi, Yutong Liu, Hiromu Horitani, Atsuki Matsukawa, Shunsuke Inoguchi, Akihiro Yoshimura, Yuki Horibe, Nesrine Sassi, Yohei Okuda, Yu Ishizuya, Takuji Hayashi, Yoshiyuki Yamamoto, Taigo Kato, Atsunari Kawashima, Norio Nonomura

**Affiliations:** ^1^ Department of Urology The University of Osaka Graduate School of Medicine Osaka Japan

**Keywords:** androgen antagonists, myosteatosis, prognosis, prostate cancer, sarcopenia

## Abstract

**Objectives:**

Prostate cancer prognostication has traditionally emphasized tumor‐related factors (e.g., Gleason score and metastatic burden). Emerging evidence suggests that physical (host) factors influence outcomes. This study evaluated whether physical factors, including myosteatosis and sarcopenia, are prognostic factors in patients with androgen receptor signaling inhibitor‐naïve, castration‐resistant prostate cancer.

**Methods:**

We retrospectively analyzed 117 androgen receptor signaling inhibitor‐naïve patients with castration‐resistant prostate cancer who received enzalutamide treatment at The University of Osaka Hospital between 2014 and 2024. Myosteatosis and sarcopenia were derived from pretreatment abdominal computed tomography. The primary endpoint was failure‐free survival. Multivariate Cox proportional hazards analysis was performed to identify independent prognostic factors.

**Results:**

Myosteatosis and sarcopenia were present in 49 (42%) and 46 (39%) patients, respectively. In univariate analyses, the Charlson comorbidity index, body mass index, and sarcopenia demonstrated no significant association with failure‐free survival, whereas myosteatosis and distant metastasis were associated with a shorter failure‐free survival. In multivariable analysis, myosteatosis remained an independent predictor of shorter failure‐free survival (hazard ratio 1.91, 95% confidence interval 1.22–2.99, *p* < 0.01). The median failure‐free survival was 6 and 17 months with and without myosteatosis, respectively (*p* < 0.01).

**Conclusions:**

Myosteatosis is an independent failure‐free survival predictor in patients with androgen receptor signaling inhibitor‐naïve castration‐resistant prostate cancer treated with enzalutamide. Computed tomography‐based evaluation of muscle quality may be useful for predicting the outcome of androgen receptor signaling inhibitors in patients with castration‐resistant prostate cancer.

**Abbreviations**
ADT: androgen deprivation therapy
AR: androgen receptor
ARSIs: androgen receptor signaling inhibitors
BMI: body mass index
CCI: charlson comorbidity index
CI: confidence interval
CRPC: castration‐resistant prostate cancer
CT: computed tomography
ECOG‐PS: Eastern Cooperative Oncology Group—performance status
FFS: failure‐free survival
HR: hazard ratio
HSPC: hormone‐sensitive prostate cancer
HU: hounsfield units
IGF1: insulin‐like growth factor 1
IMAT: increased intramuscular adipose tissue
IMCL: intramyocellular lipid content
IQR: interquartile range
L3: third lumbar vertebra
OS: overall survival
PCa: prostate cancer
PFS: progression‐free survival
PMI: psoas muscle index
PSA: prostate specific antigen

## 1 Introduction

Prostate cancer (PCa) is one of the most common malignancies worldwide. Despite advances in treatment, metastatic disease remains associated with a poor prognosis, with a 5‐year survival rate of only 26%–30% [1]. For metastatic PCa, both hormone‐sensitive prostate cancer (HSPC) and castration‐resistant prostate cancer (CRPC), androgen receptor signaling inhibitors (ARSIs) are crucial, with enzalutamide being among the most widely used agents.

Historically, PCa prognostication has been dominated by tumor‐related factors, such as Gleason score, disease stage, and the burden and distribution of metastatic lesions [2]. Consistently, the 5‐year analysis of the phase III PREVAIL trial in chemotherapy‐naïve mCRPC identified independent prognostic factors for overall survival (OS), including ≥ 10 bone metastases, liver metastases, and elevated alkaline phosphatase and lactate dehydrogenase [3]. Importantly, these factors are fundamentally tumor‐side factors.

Besides these tumor‐related parameters, physical frailty has recently attracted attention as a PCa prognostic factor. Notably, population‐based registry studies have shown that Japanese patients receiving primary androgen deprivation therapy (ADT) experience lower PCa‐specific mortality compared with Western populations, even after adjustment for disease‐related factors [4]. This observation suggests that host‐related factors may contribute to treatment outcomes beyond tumor characteristics alone. For instance, increased body mass index (BMI), which reflects obesity, has been associated with PCa‐specific mortality [5]. Among frailty components, sarcopenia (muscle mass loss) and myosteatosis (intramuscular fat infiltration), both of which can be noninvasively and quantitatively assessed using computed tomography (CT) imaging, are increasingly recognized as clinically relevant [6]. Sarcopenia, defined as the loss of skeletal muscle mass, is widely acknowledged as a negative prognostic factor for various malignancies [7]. Nonetheless, its prognostic significance in PCa remains controversial. For example, a meta‐analysis by Kovac et al. found no significant association between sarcopenia and PCa‐specific mortality or treatment response [8]. Conversely, myosteatosis represents qualitative muscle impairment due to intramuscular fat infiltration and has been associated with poor outcomes in multiple cancer types. A systematic review by Alexio et al. reported that myosteatosis is associated with shorter OS in several malignancies, including gastrointestinal and breast cancers [9]. However, studies on the association between myosteatosis and prognosis in PCa are limited, and its role in patients with CRPC undergoing ARSI therapy has not been evaluated.

This study aimed to evaluate whether physical (host) factors, including myosteatosis and sarcopenia, can be prognostic markers in patients with ARSI‐naïve CRPC treated with enzalutamide. This study will provide new insights into the prognostic value of muscle quality assessment via CT for patients with CRPC.

## 2 Methods

### 2.1 Study Cohort

**FIGURE 1:**
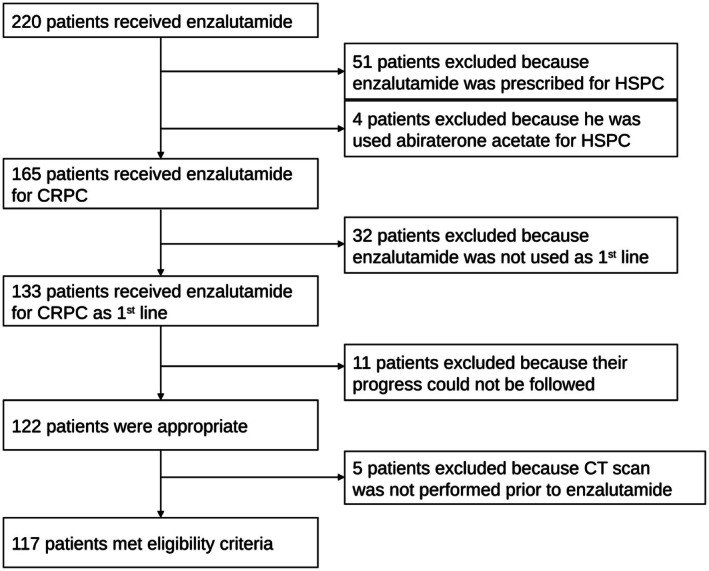
Study schematic. CRPC: Castration‐resistant prostate cancer, CT: Computed tomography, HSPC: Hormone‐sensitive prostate cancer.

This study builds on a previously analyzed cohort [10] by expanding the number of cases and extending the observation period. We retrospectively reviewed patients with ARSI‐naïve CRPC treated with enzalutamide as first‐line therapy at The University of Osaka Hospital between June 2014 and December 2024. Patients who were treated with enzalutamide or abiraterone during the HSPC stage were excluded. However, only ARSI‐naïve patients who received docetaxel therapy during HSPC were included. Ultimately, 117 patients met the inclusion criteria and underwent CT evaluation before enzalutamide initiation (Figure 1).

### 2.2 Data Collection

CRPC and prostate specific antigen (PSA) progression were defined according to the previously established criteria [11]. The following data were retrospectively collected from the electronic medical records: age, BMI, Gleason score, initial PSA, clinical laboratory values, imaging data at the start of enzalutamide treatment, and comorbidities.

### 2.3 Definition of Myosteatosis, Sarcopenia, and Charlson Comorbidity Index

**FIGURE 2:**
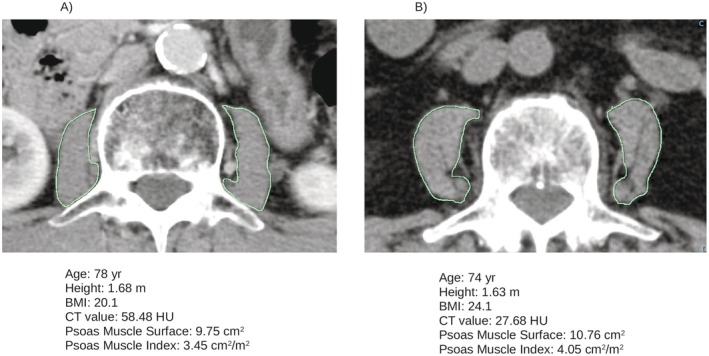
Measurement of the area and density of each psoas muscle on the axial non‐contrast computed tomography scan at level L3. Case A: A patient with sarcopenia but without myosteatosis. The FFS with enzalutamide treatment was 20 months. Case B: A patient with sarcopenia and myosteatosis. The FFS with enzalutamide was 3 months. BMI: Body mass index, FFS: Failure‐free survival.

Quantitative analysis of the psoas muscle was conducted to assess the muscle area and density, expressed in Hounsfield Units (HU). CT imaging was performed using 64‐slice or higher multidetector CT scanners available at our institution. A digital free‐hand manual outline of the left and right psoas muscles was created on axial non‐contrast CT images at the mid‐level of the third lumbar vertebra (L3). The cross‐sectional area and mean CT attenuation values for each muscle were computed automatically (Figure 2). To assess skeletal muscle mass, the psoas muscle index (PMI; cm^2^/m^2^) was calculated by dividing the total psoas muscle area by the square of the patient's height (m). Muscle quality was evaluated using the average CT attenuation value of bilateral psoas muscles. Sarcopenia was defined as a PMI < 4.24 cm^2^/m^2^ based on the previously reported cutoff value [12]. Moreover, myosteatosis was determined as a mean CT attenuation value of the psoas muscle at the L3 level of less than 41 HU in patients with BMI < 25, and less than 33 HU in those with BMI ≥ 25, based on previously established thresholds [9].

The comorbidity burden was evaluated using the Charlson comorbidity index (CCI) based on established criteria [13].

### 2.4 Endpoints

The primary endpoint was failure‐free survival (FFS), which was defined as the time from the initiation of enzalutamide treatment to the first of the following events: clinical progression, PSA progression, or radiographic progression, according to the Prostate Cancer Working Group 3 criteria [11]. Besides estimating survival outcomes, we examined the association between FFS and tumor‐related and host (physical) factors. The secondary endpoints were PSA‐progression‐free survival (PFS) and OS.

### 2.5 Statistical Analysis

Survival curves were generated using the Kaplan–Meier method, and intergroup comparisons were performed using the log‐rank test. Associations between clinical variables, FFS, and PSA‐PFS were assessed using Cox proportional hazards models. Statistical significance was defined as a two‐sided *p* < 0.05. All statistical analyses were performed using the JMP Pro software (SAS Institute Inc., Cary, NC, USA).

## 3 Results

### 3.1 Patient Characteristics

The baseline characteristics of the 117 patients are summarized in Table 1. The median patient age was 75 years (interquartile range [IQR] 69–81 years). At diagnosis, 23 patients (20%) had a Gleason score of ≥ 8. Regarding metastatic status, distant metastases were observed in 92 patients (79%). Seven patients (6%) received upfront docetaxel during the mHSPC phase.

**TABLE 1:** Patient characteristics.

	Total (*n* = 117)	Myosteatosis (*n* = 49)	Non‐myosteatosis (*n* = 68)	*p*
Median age, years (IQR)	75 (69–81)	76 (71–84)	73 (66–81)	< 0.01
ECOG‐PS				0.39
0	95 (81)	38 (78)	57 (84)	
≥ 1	22 (19)	11 (22)	11 (16)	
Gleason sum at diagnosis, *n* (%)				0.15
< 8	23 (20)	6 (12)	17 (25)	
≥ 8	84 (72)	38 (78)	46 (68)	
Not available	10 (9)	5 (10)	5 (7)	
Treatment for HSPC, *n* (%)				0.68
CAB	76 (65)	30 (61)	46 (68)	
ADT	33 (28)	16 (33)	17 (25)	
Upfront docetaxel	7 (6)	3 (6)	4 (6)	
Other	1 (1)	0 (0)	1 (1)	
Median time to CRPC, months (IQR)	19 (10–36)	23 (11–50)	17 (9–29)	0.16
BMI, kg/m^2^ (IQR)	22.2 (20.2–24.9)	21.4 (20.2–24.6)	22.8 (20.3–25)	0.52
Psoas muscle index, cm^2^/m^2^ (IQR)	4.54 (3.90–5.41)	4.70 (3.90–5.65)	4.40 (3.90–5.03)	0.33
Sarcopneia, *n* (%)				0.29
Yes	46 (39)	22 (45)	24 (35)	
No	71 (61)	27 (55)	44 (65)	
Charlson comorbidity index, *n*(%)				0.85
2–5	64 (55)	26 (53)	38 (56)	
≥ 6	53 (45)	23 (47)	30 (44)	
Median serum initial PSA level at diagnosis, ng/mL (IQR)	32.8 (12.4–159.6)	53 (12.6–211.7)	29.8 (11.6–81.0)	0.52
Lymph node metastasis before ENZ, *n* (%)				0.53
Yes	32 (27)	15 (31)	17 (25)	
No	81 (69)	32 (65)	49 (72)	
Not available	4 (3)	2 (4)	2 (3)	
Bone metastasis before ENZ, *n* (%)				0.31
Yes	79 (68)	36 (73)	43 (63)	
No	35 (30)	12 (24)	23 (34)	
Not available	3 (3)	1 (2)	2 (3)	
Visceal metastasis before ENZ, *n* (%)				0.77
Yes	13 (11)	6 (12)	7 (10)	
No	100 (85)	41 (84)	59 (87)	
Not available	4 (3)	2 (4)	2 (3)	
Distant metastasis^a^ before ENZ, *n* (%)				0.05
Yes	92 (79)	43 (88)	49 (72)	
No	22 (19)	5 (10)	17 (25)	
Not available	3 (3)	1 (2)	2 (3)	
Myosteatosis at diagnosis, *n* (%)				< 0.01
Yes	13 (11)	11 (22)	2 (3)	
No	85 (73)	32 (65)	53 (78)	
Not available	19 (16)	6 (12)	13 (29)	

Abbreviations: ADT, androgen deprivation therapy; BMI, body mass index; CAB, combined androgen blockage; CRPC, castration‐resistant prostate cancer; ECOG‐PS, Eastern Cooperative Oncology Group – performance status; ENZ, enzalutamide; HSPC, hormone‐sensitive prostate cancer; IQR, interquartile range; PSA, prostate specific antigen.

^a^ Distant metastasis is defined as the presence of at least one of the following: distant lymph node metastasis, bone metastasis, or visceral metastasis.

Regarding physical factors, myosteatosis was present in 49 patients (42%), consistent with the findings of previous systematic reviews [8]. The median PMI was 4.54 cm^2^/m^2^ (IQR 3.90–5.41). Based on a PMI cutoff of 4.24 cm^2^/m^212^, 46 patients (39%) were classified as having sarcopenia. The median BMI was 22.2. Regarding the CCI, 53 patients (45%) had a score of 6 or higher. Regarding Eastern Cooperative Oncology Group—performance status (ECOG‐PS), 95 (81%) had PS of 0. The median time to CRPC was 19 months (IQR 10–36 months), and the median observation period was 27 months (IQR 15–46 months).

When patients were stratified according to the presence or absence of myosteatosis, those with myosteatosis were significantly older than those without myosteatosis (76 vs. 73 years, *p* < 0.01). In addition, the prevalence of distant metastasis before enzalutamide initiation tended to be higher in patients with myosteatosis (43/48 [88%] vs. 49/68 [72%], *p* = 0.05). A significant difference was also observed in the prevalence of myosteatosis at treatment initiation (11/48 [22%] vs. 2/68 [3%], *p* < 0.01). Other baseline clinical and tumor‐related characteristics were comparable between the two groups.

### 3.2 Identification of Prognostic Factors

In the univariate analysis of FFS, myosteatosis (hazard ratio [HR] 1.95, 95% confidence interval [CI] 1.26–3.02, *p* < 0.01) was significantly associated with shorter FFS. Conversely, other physical factors such as ECOG‐PS, CCI, BMI, and sarcopenia exhibited no significant associations. Among tumor‐associated factors, distant metastasis (HR 1.99, 95% CI 1.07–3.70, *p* = 0.03) was also associated with shorter FFS.

In the multivariate model 1 including variables that were significant in the univariate analysis (myosteatosis and distant metastasis), only myosteatosis remained an independent predictor of FFS (HR 1.91, 95% CI 1.22–2.99, *p* < 0.01). In a multivariate model 2 additionally adjusted for age, which differed significantly between the myosteatosis and non‐myosteatosis groups, myosteatosis remained independently associated with shorter FFS (HR 1.98, 95% CI 1.26–3.11, *p* < 0.01) (Table 2).

**TABLE 2:** Univariable and multivariable analyses for FFS.

Variable	Vs. referent	Univariable		Multivariable model 1		Multivariable model 2	
		HR (95% CI)	*p*	HR (95% CI)	*p*	HR (95% CI)	*p*
Age	< 75 vs. ≥ 75 years	1.51 (0.98–2.35)	0.06			1.55 (0.99–2.42)	0.06
ECOG PS	≥ 1 vs. < 1	1.74 (1.00–3.03)	0.05				
Initial PSA	High vs. low	1.13 (0.74–1.75)	0.58				
Gleason score at diagnosis	≥ 8 vs. < 8	1.12 (0.63–1.98)	0.69				
Charlson comorbidity index	≥ 6 vs. < 6	1.05 (0.62–1.49)	0.87				
Time to CRPC	< 19 vs. ≥ 19 months	1.49 (0.95–2.35)	0.09				
Distant metastasis before ENZ	Present vs. absent	1.99 (1.07–3.70)	0.03	1.84 (0.98–3.42)	0.06	1.83 (0.98–3.40)	0.06
BMI	≥ 22 vs. < 22	1.01 (0.70–1.68)	0.7				
Sarcopenia	Present vs. absent	0.82 (0.52–1.27)	0.37				
Myosteatosis before ENZ	Present vs. absent	1.95 (1.26–3.02)	< 0.01	1.91 (1.22–2.99)	< 0.01	1.98 (1.26–3.11)	< 0.01

Abbreviations: BMI, body mass index; CI, confidence interval; CRPC, castration‐resistant prostate cancer; ECOG‐PS, Eastern Cooperative Oncology Group – performance status; ENZ, enzalutamide; HR, hazard ratio; PSA, prostate specific antigen.

The univariate analysis identified age, ECOG‐PS, time to CRPC diagnosis, distant metastasis, and myosteatosis as significant predictors of PSA‐PFS. Multivariate analysis confirmed myosteatosis (HR 1.83, 95% CI 1.14–2.93, *p* = 0.01) as an independent prognostic factor. Similar to FFS, other physical factors, including BMI, sarcopenia, and CCI, were not associated with PSA‐PFS (Table S1).

### 3.3 Impact of Myosteatosis on FFS and OS

**FIGURE 3:**
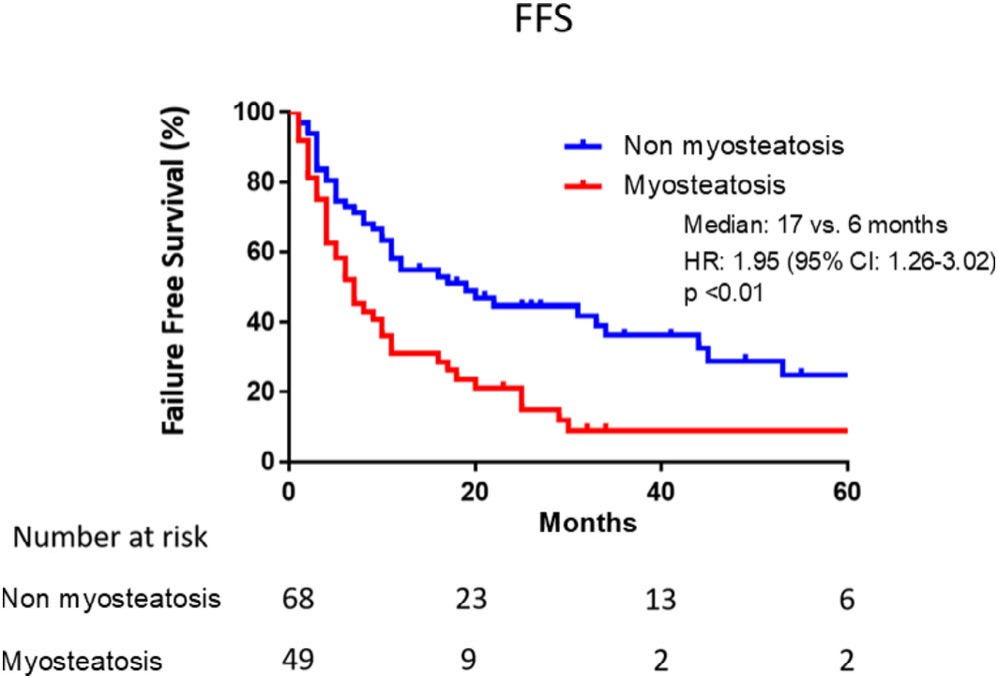
Kaplan–Meier curves showing failure‐free survival stratified by the presence or absence of myosteatosis. CI: Confidence interval, FFS: Failure‐free survival, HR: Hazard ratio.

Kaplan–Meier analysis of FFS is displayed in Figure 3. The median FFS was significantly shorter in patients with myosteatosis (6 months) than in those without it (17 months; *p* < 0.01). Similarly, Kaplan–Meier analysis of PSA‐PFS demonstrated a median of 7 months in the myosteatosis group versus 19 months in the non‐myosteatosis group (Figure S1).

A median OS of 38 months and 59 months was observed in patients with and without myosteatosis respectively, but OS did not differ significantly between the two groups (*p* = 0.23; Figure S2). Post‐enzalutamide treatment patterns stratified by the presence or absence of myosteatosis are illustrated using the river plots (Figure S3). In both groups, docetaxel was the most frequently used second‐line treatment after enzalutamide; however, patients subsequently followed diverse treatment courses, reflecting heterogeneity in post‐enzalutamide management.

To clarify the impact of myosteatosis at the initiation of PCa treatment, we performed additional analyses focusing on the presence of myosteatosis before hormonal therapy and its association with prognosis. At the initiation of PCa treatment, 13 patients had myosteatosis, whereas 85 patients did not (Table 1). The presence of myosteatosis at the initiation of PCa treatment was not significantly associated with either FFS or OS after enzalutamide initiation (Figure S4). Furthermore, no significant differences in FFS or OS were observed between patients with myosteatosis already present before hormonal therapy (*n* = 11) and those who developed myosteatosis during treatment (*n* = 32) (Figure S5). These findings suggest that myosteatosis status at the initiation of enzalutamide treatment is more strongly associated with treatment outcomes than myosteatosis status at the initiation of PCa treatment.

## 4 Discussion

In this ARSI‐naïve CRPC cohort, myosteatosis was identified as an independent prognostic factor significantly associated with a shorter FFS following enzalutamide initiation. Myosteatosis retained independent prognostic value in multivariable models for both FFS and PSA‐PFS, underscoring the clinical relevance of muscle quality beyond traditional tumor‐related factors. Conversely, sarcopenia showed no significant association with FFS in this cohort, supporting the notion that muscle quality may be more informative than muscle quantity for prognostic prediction in CRPC.

Clinical studies on myosteatosis in PCa are limited. Lee et al. analyzed 411 patients with CRPC and found a strong association between myosteatosis and shorter OS (median 15 vs. 26 months, *p* < 0.001) [14]. Xu et al. evaluated 182 patients with metastatic PCa including CRPC and mHSPC and observed a significant association between myosteatosis and shorter OS in the univariate analysis (HR 7.18, *p* = 0.007), which was not maintained in the multivariate analysis (HR 0.97, *p* = 0.06) [15]. Furthermore, Sheean et al. reported that, in patients with mHSPC, prior to hormone therapy, myosteatosis was associated with shorter OS (HR 2.34, *p* = 0.09), with a stronger trend among Black patients (HR 4.39, 95% CI 0.92–21.1, *p* = 0.06) [16]. In contrast, our study focused on patients with CRPC who underwent ARSI therapy, providing new insights into the association between myosteatosis and prognosis in PCa.

Obesity and myosteatosis represent distinct pathological entities with different biological characteristics. Obesity is characterized by the excessive accumulation of subcutaneous and visceral fat, reflecting generalized weight gain, whereas myosteatosis is defined by impaired muscle quality due to ectopic fat redistribution within skeletal muscle, particularly through increased intramuscular adipose tissue (IMAT) and intramyocellular lipid content (IMCL) [17]. Several biological mechanisms have been proposed to explain the role of myosteatosis in tumor progression. First, intramuscular fat infiltration reduces myokine secretion, diminishing the inhibitory effects of these factors on tumor proliferation and invasion [18]. Second, aging and therapeutic interventions induce chronic secretion of inflammatory cytokines such as interleukin‐6 (IL6), tumor necrosis factor‐α, and interleukin‐1β, promoting a pro‐inflammatory microenvironment that facilitates tumor progression [19]. Third, IMAT and IMCL accumulation cause insulin resistance and hyperinsulinemia [20], which may enhance tumor growth via activation of the insulin‐like growth factor 1 (IGF1)–IGF1 receptor (IGF1R) pathway [21]. Notably, Xu et al. reported that even among patients with high BMI, those without myosteatosis had a better prognosis than those with myosteatosis [15]. This finding supports the notion that obesity and myosteatosis are distinct pathological conditions with different biological and prognostic implications in PCa.

In this study, myosteatosis was an independent predictor of FFS following enzalutamide initiation. The reduced efficacy of enzalutamide in patients with myosteatosis may be partly explained by inflammatory and metabolic signaling pathways, particularly the IL6 and IGF1 pathways. Tumor‐derived IL6 has been shown to induce myosteatosis through mechanisms distinct from simple obesity [22], and activation of the IL6–STAT3 pathway has been implicated in enzalutamide resistance in PCa models [23]. Consistent with this, our previous study identified elevated serum IL6 as a poor prognostic factor in patients treated with enzalutamide [24]. As discussed above, myosteatosis is also associated with increased insulin resistance, which may lead to activation of the IGF1 signaling pathway. Similarly, IGF‐mediated phosphorylation of androgen receptor (AR) may enhance AR signaling, potentially attenuating enzalutamide efficacy [25]. Regardless of these mechanisms, this study found that patients with myosteatosis tended to have shorter OS, though this difference was not statistically significant. This may reflect the heterogeneity of treatment regimens following enzalutamide therapy.

In addition, ADT alters skeletal muscle metabolism and promotes intramuscular fat deposition, thereby contributing to the development of myosteatosis [26]. Prolonged ADT may cause myosteatosis and worsen clinical outcomes. In the present study, a small proportion of patients received upfront docetaxel during the metastatic HSPC phase; however, the number of such cases was limited, and their impact on muscle quality was considered minimal within this cohort. As upfront treatment strategies—including intensified systemic therapies—continue to expand in clinical practice, longer exposure to ADT may increasingly predispose patients to the development of myosteatosis, raising concerns regarding its clinical implications. However, myosteatosis is a potentially preventable condition. Although evidence from long‐term clinical studies on nutritional interventions in humans remains limited, exercise interventions have been shown to significantly reduce intramuscular fat accumulation. Ramirez‐Velez et al. demonstrated in a systematic review that exercise improves muscle fat infiltration, likely through enhanced mitochondrial function and fatty acid oxidation capacity within skeletal muscle [27]. Furthermore, a relatively brief exposure to exercise significantly improved muscle mass, strength, and physical function in patients with PCa receiving ADT [28]. Although direct evidence for the reversibility of myosteatosis is lacking, lifestyle modifications and exercise may improve the prognosis of patients with PCa undergoing ADT.

This study had some limitations. First, this was a retrospective, single‐center analysis with a relatively small sample size. Second, although CT‐based metrics were used to assess sarcopenia and myosteatosis, universally standardized cutoffs and definitions have not yet been established, which may limit the external validity and precision of the results. Prospective multicenter validation with larger cohorts and methodological standardization is warranted.

Nevertheless, our data reveal that myosteatosis is independently associated with shorter FFS following enzalutamide initiation in patients with ARSI‐naïve CRPC. Quantitative assessment of intramuscular fat on pretreatment CT is simple and reproducible. Importantly, this association does not imply that enzalutamide should be avoided in patients with myosteatosis. This is because myosteatosis is also associated with reduced survival rates in alternative taxane‐based therapies [29, 30]. Rather, because myosteatosis is a potentially modifiable condition—even during ADT [28], its identification may help recognize patients who could benefit from concomitant supportive interventions, such as nutritional support and structured exercise programs, while continuing enzalutamide treatment. From this perspective, myosteatosis may serve not only as a marker of earlier treatment failure but also as a clinically actionable host‐related factor that informs individualized supportive care strategies during ARSI therapy.

## Author Contributions

Tomohiro Kanaki: writing – original draft; investigation; visualization. Koji Hatano: conceptualization, writing – review and editing; investigation. Toshiki Oka: investigation. Masaru Tani: investigation. Masatoshi Konishi: investigation. Yutong Liu: investigation. Yuki Horitani: investigation. Atsuki Matsukawa: investigation. Shunsuke Inoguchi: investigation. Akihiro Yoshimura: investigation. Yuki Horibe: investigation. Nesrine Sassi: investigation. Yohei Okuda: investigation. Yu Ishizuya: investigation. Takuji Hayashi: investigation. Yoshiyuki Yamamoto: writing – review and editing, Taigo Kato: investigation. Atsunari Kawashima: investigation. Norio Nonomura: writing – review and editing; supervision.

## Ethics Statement

This study has been approved by the University of Osaka Ethics Committee (Approval Number: 1339723) and conducted in accordance with the principles of the Declaration of Helsinki.

## Consent

We obtained written informed consent from all participants.

## Conflicts of Interest

Kentaro Takezawa and Norio Nonomura are Editorial Board members of the International Journal of Urology and co‐authors of this article. To minimize bias, they were excluded from all editorial decision‐making related to the acceptance of this article for publication. All other authors declare no conflicts of interest.

## Supporting information


**Figure S1:** Kaplan–Meier curves showing PSA‐PFS stratified by the presence or absence of myosteatosis.
**Figure S2:** Kaplan–Meier curves showing OS stratified by the presence or absence of myosteatosis.
**Figure S3:** Post‐enzalutamide treatment flow stratified by the presence or absence of myosteatosis.
River plots illustrate subsequent treatment sequences after enzalutamide initiation in patients with myosteatosis (A) and without myosteatosis (B). Node width represents the number and proportion of patients within each treatment category.
**Figure S4:** Kaplan–Meier curves showing survival outcomes after enzalutamide initiation stratified by the presence or absence of myosteatosis before hormonal therapy.
(A) Failure‐free survival (FFS). (B) Overall survival (OS).
**Figure S5:** Kaplan–Meier curves showing survival outcomes after enzalutamide initiation among patients with myosteatosis at the time of enzalutamide initiation, stratified by the presence or absence of myosteatosis before hormonal therapy.
(A) Failure‐free survival (FFS). (B) Overall survival (OS).

**TABLE S1:** Univariable and multivariate analyses for PSA‐PFS.

## Data Availability

The data that support the findings of this study are available from the corresponding author upon reasonable request.
