# Supplementary material for: Myosteatosis Predicts Poor Prognosis in Patients With Castration‐Resistant Prostate Cancer Treated With Enzalutamide
Source: Int J Urol. 2026 Apr 29;33:e70476. doi: 10.1111/iju.70476 (PMC13127351; doi:10.1111/iju.70476)
Supplement: Supplementary file 1 — Figure S1: Kaplan–Meier curves showing PSA‐PFS stratified by the presence or absence of myosteatosis. Figure S2: Kaplan–Meier curves showing OS stratified by the presence or absence of myosteatosis. Figure S3: Post‐enzalutamide treatment flow stratified by the presence or absence of myosteatosis. River plots illustrate subsequent treatment sequences after enzalutamide initiation in patients with myosteatosis (A) and without myosteatosis (B). Node width represents the number and proportion of patients within each treatment category. Figure S4: Kaplan–Meier curves showing survival outcomes after enzalutamide initiation stratified by the presence or absence of myosteatosis before hormonal therapy. (A) Failure‐free survival (FFS). (B) Overall survival (OS). Figure S5: Kaplan–Meier curves showing survival outcomes after enzalutamide initiation among patients with myosteatosis at the time of enzalutamide initiation, stratified by the presence or absence of myosteatosis before hormonal therapy. (A) Failure‐free survival (FFS). (B) Overall survival (OS). [file IJU-33-0-s001.pdf]

Supplementary Figure 1

Kaplan–Meier curves showing PSA-PFS stratified by the presence or absence of myosteatosis.

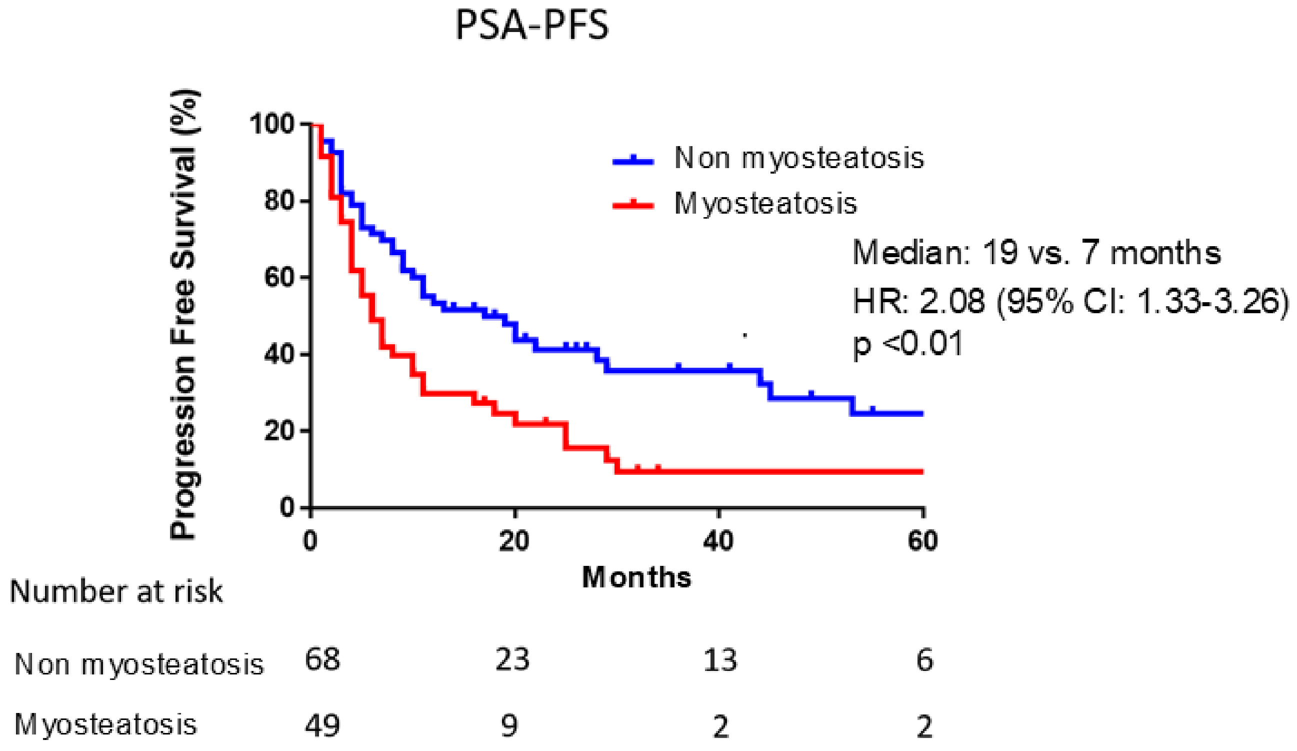

CI: confidence interval, HR: hazard ratio, PSA: prostate-specific antigen, PFS: progression-free survival.

Supplementary Figure 2

Kaplan–Meier curves showing OS stratified by the presence or absence of myosteatosis.

OS

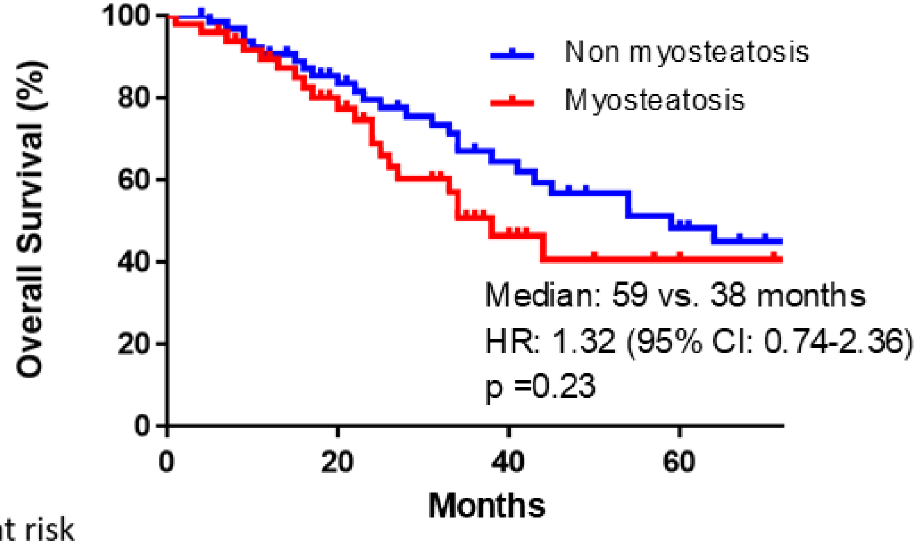

Number at risk

Non myosteatosis	68	46	27	17
Myosteatosis	49	29	11	5

CI: confidence interval, HR: hazard ratio, OS: overall survival.

Supplementary Figure 3

Post-enzalutamide treatment flow stratified by the presence or absence of myosteatosis.

River plots illustrate subsequent treatment sequences after enzalutamide initiation in patients with myosteatosis (A) and without myosteatosis (B). Node width represents the number and proportion of patients within each treatment category.

A)

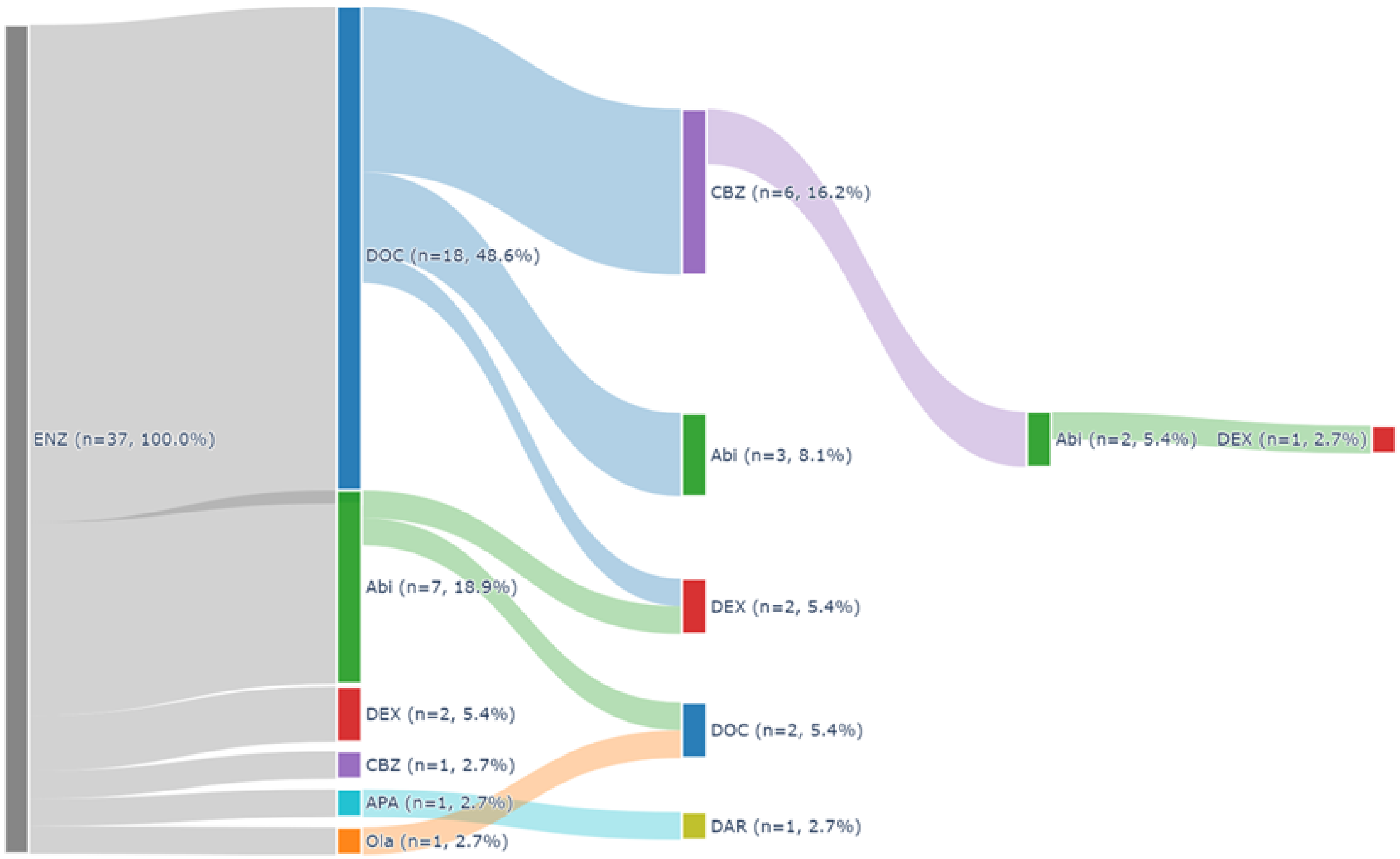

B)

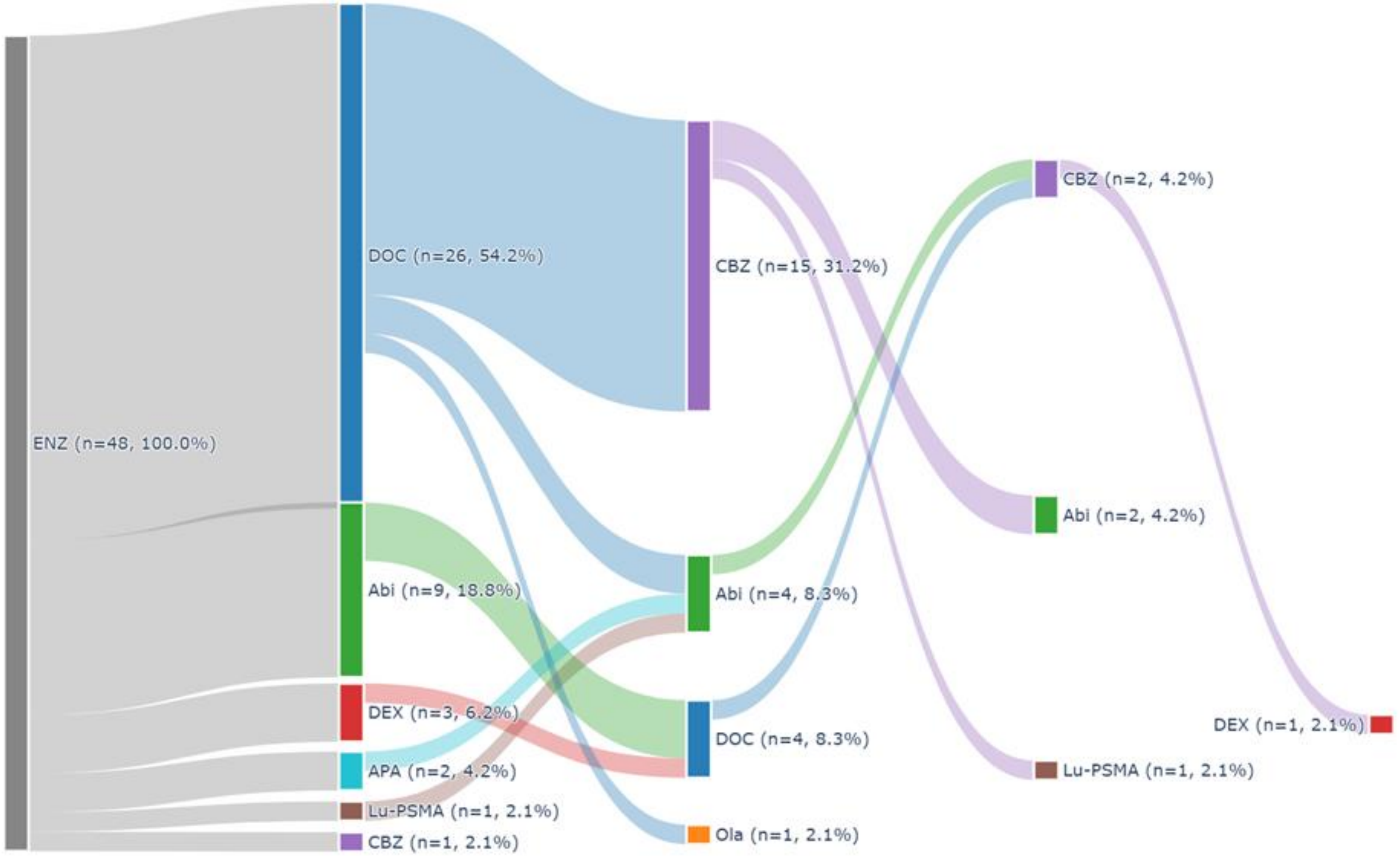

Abi: abiraterone, APA: apalutamide, CBZ: cabazitaxel, DAR; darolutamide, DEX: dexamethasone, DOC: docetaxel, ENZ: enzalutamide, Lu: lutetium, Ola: olaparib, PSMA: prostate-specific membrane antigen.

Supplementary Figure 4

Kaplan–Meier curves showing survival outcomes after enzalutamide initiation stratified by the presence or absence of myosteatosis before hormonal therapy.
(A) Failure-free survival (FFS). **(B)** Overall survival (OS).

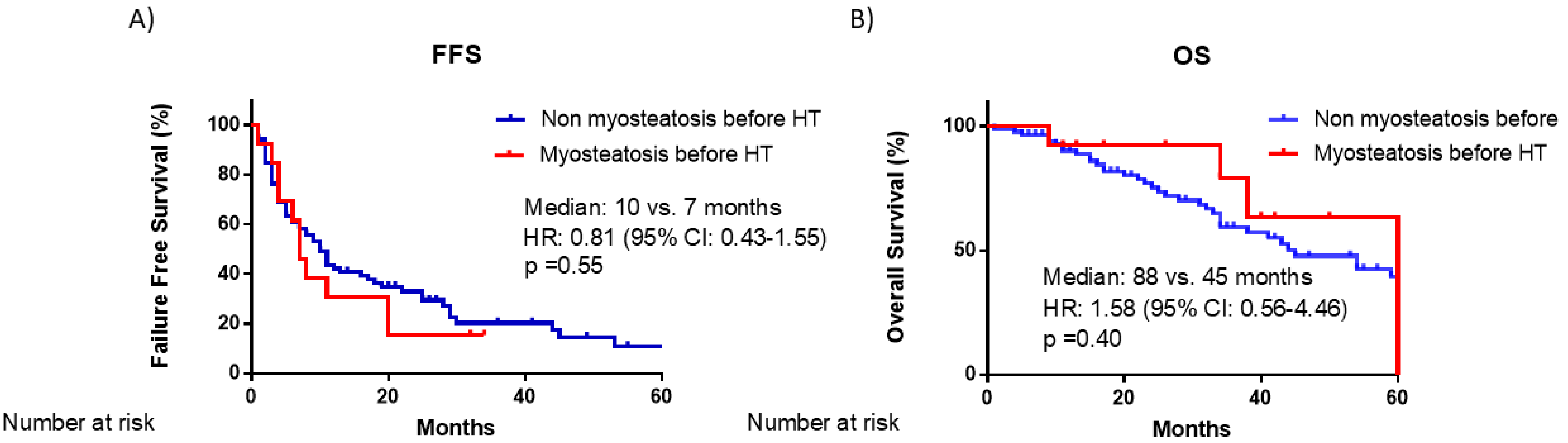

A) Number at risk

Non myosteatosis before HT	85	21	9	3
Myosteatosis before HT	13	4	1	1

B) Number at risk

Non myosteatosis before HT	85	54	27	14
Myosteatosis before HT	13	9	4	2

CI: confidence interval, FFS; Failure-free survival, HR: hazard ratio, HT: hormonal therapy, OS: overall survival.

Supplementary Figure 5

Kaplan–Meier curves showing survival outcomes after enzalutamide initiation among patients with myosteatosis at the time of enzalutamide initiation, stratified by the presence or absence of myosteatosis before hormonal therapy.
(A) Failure-free survival (FFS). (B) Overall survival (OS).

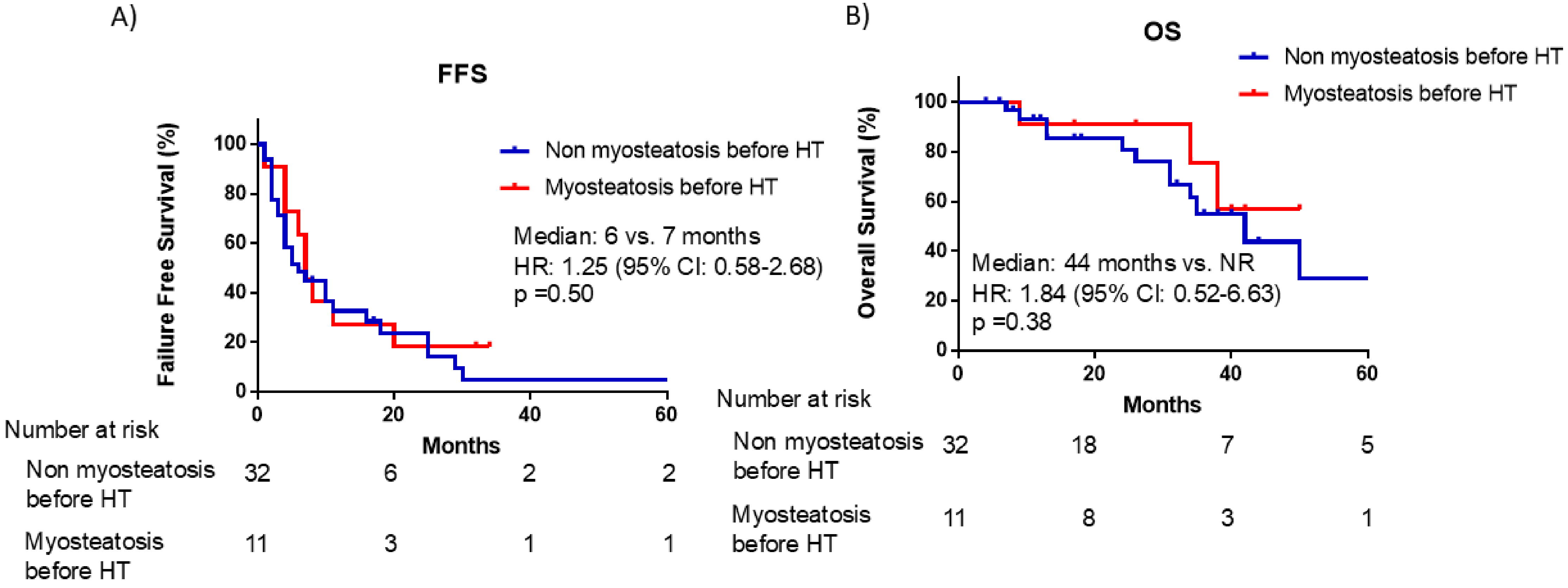

CI: confidence interval, FFS; Failure-free survival, HR: hazard ratio, HT: hormonal therapy, NR: not reached, OS: overall survival.
